# Surgical Shock and Some Related Problems

**Published:** 1918-10

**Authors:** 


### ABSTRACTS

### SURGERY

***Surgical Shock and Some Related Problems.*** By J. E. Sweet. *American Journal of the Medical Sciences.* Philadelphia, May, 1918, Vol. CLV, No. 5, pp. 625-639.

Sweet says there is a tendency to dislocate physiology from clinical medicine, and makes emphatic the point that a surgeon must know as much as any man in any branch of medicine.

He defines shock as a bleeding to death in the venous system of the body; that is, that in shock the blood collects in the veins, chiefly of the splanchnic system, and the patient dies because the blood fails to reach the right heart. The role played in the condition of shock by hydrostatic pressure is not clear, but it is a actor which cannot be ignored. What we commonly call blood pressure is the tension of the vessel wall produced by the systole of the ventricle and transmitted by an incompressible fluid.

He defines syncope as a condition in which the blood pressure falls because the great automatic centers are cut off short. Collapse is defined by him as a condition in which, by the phenomenon called cardiac inhibition, the machinery of the circulation falls. Here vasomotor stimulation is not indicated; saline infusion, bandaging of the extremities, perfusion of the blood — all this is contra-indicated; the engine is stalled, and putting on more load will not start it. In fact it is not unlikely that a venesection would be of more help in this condition than any effort which would increase the burden of the heart. Concerning hemorrhage he says, "without denying that loss of blood may be a factor in the production of the shock, which appeals to my mind as true surgical shock, I still object to grouping deaths due to hemorrhage under the caption of deaths due to shock. Death occurs after hemorrhage and because the patient bleeds to death".

He defines true surgical shock as a condition marked by a gradual, persistent, progressive fall of blood pressure, such as characterizes certain cases after extensive crush injury with practically no loss of blood, cases of extensive burns, and many intra-abdominal conditions; these are, in his opinion, instances of primary failure of the peripheral vasomotor mechanism and are associated in some manner with a disturbance of adrenal function. By experimentation the condition which compares with surgical shock can be produced by the complete removal of both adrenals. From the practical side adrenalin has proved valuable in the treatment of this condition.

His ideas of shock are entirely compatible with the treatment of Porter (*Boston Medical and Surgical Journal*, 1916). Porter, as a result of his studies of shock at the front, advises :

1. A special position of the wounded so that the abdominal vessels shall be higher than the heart and brain (in other words, counteract the effect of hydrostatic pressure).
2. Heat. (The use of heat in shock is not clear to me. It seems like the empirical combating of a symptom, but is universally used.)
3. Intravenous injection of saline solution.
4. Intravenous injection of epinephrin.
5. The transfusion of blood in certain cases.
6. The observation of the diastatic pressure every half hour, as an index of the condition of the patient.

